# Whole‐genome sequencing identifies *interferon-induced protein IFI6/IFI27-like* as a strong candidate gene for VNN resistance in European sea bass

**DOI:** 10.1186/s12711-023-00805-2

**Published:** 2023-05-04

**Authors:** Emilie Delpuech, Marc Vandeputte, Romain Morvezen, Anastasia Bestin, Mathieu Besson, Joseph Brunier, Aline Bajek, Boudjema Imarazene, Yoannah François, Olivier Bouchez, Xavier Cousin, Charles Poncet, Thierry Morin, Jean-Sébastien Bruant, Béatrice Chatain, Pierrick Haffray, Florence Phocas, François Allal

**Affiliations:** 1 MARBEC, Univ. Montpellier, CNRS, Ifremer, IRD, INRAE, 34250 Palavas-Les-Flots, France; 2 Université Paris-Saclay, INRAE, AgroParisTech, GABI, 78350 Jouy-en-Josas, France; 3 SYSAAF, Station LPGP/INRAE, Campus de Beaulieu, 35042 Rennes, France; 4 Ecloserie Marine de Gravelines-Ichtus, Gloria Maris Group, 59273 Gravelines, France; 5 Fermes Marines Du Soleil, 17840 La Brée Les Bains, France; 6 ANSES, Unit Virology, Immunology and Ecotoxicology of Fish, Technopôle Brest-Iroise, 29280 Plouzané, France; 7 US 1426, GeT-PlaGe, INRAE, Genotoul, Castanet-Tolosan, France; 8 INRAE-UCA, UMR 1095 GDEC, 63000 Clermont-Ferrand, France

## Abstract

**Background:**

Viral nervous necrosis (VNN) is a major disease that affects European sea bass, and understanding the biological mechanisms that underlie VNN resistance is important for the welfare of farmed fish and sustainability of production systems. The aim of this study was to identify genomic regions and genes that are associated with VNN resistance in sea bass.

**Results:**

We generated a dataset of 838,451 single nucleotide polymorphisms (SNPs) identified from whole-genome sequencing (WGS) in the parental generation of two commercial populations (A: 2371 individuals and B: 3428 individuals) of European sea bass with phenotypic records for binary survival in a VNN challenge. For each population, three cohorts were submitted to a red-spotted grouper nervous necrosis virus (RGNNV) challenge by immersion and genotyped on a 57K SNP chip. After imputation of WGS SNPs from their parents, quantitative trait loci (QTL) were mapped using a Bayesian sparse linear mixed model (BSLMM). We found several QTL regions that were specific to one of the populations on different linkage groups (LG), and one 127-kb QTL region on LG12 that was shared by both populations and included the genes *ZDHHC14,* which encodes a palmitoyltransferase, and *IFI6/IFI27-like*, which encodes an interferon-alpha induced protein. The most significant SNP in this QTL region was only 1.9 kb downstream of the coding sequence of the *IFI6/IFI27-like* gene. An unrelated population of four large families was used to validate the effect of the QTL. Survival rates of susceptible genotypes were 40.6% and 45.4% in populations A and B, respectively, while that of the resistant genotype was 66.2% in population B and 78% in population A.

**Conclusions:**

We have identified a genomic region that carries a major QTL for resistance to VNN and includes the *ZDHHC14* and *IFI6/IFI27-like* genes. The potential involvement of the interferon pathway, a well-known anti-viral defense mechanism in several organisms (chicken, human, or fish), in survival to VNN infection is of particular interest. Our results can lead to major improvements for sea bass breeding programs through marker-assisted genomic selection to obtain more resistant fish.

**Supplementary Information:**

The online version contains supplementary material available at 10.1186/s12711-023-00805-2.

## Background

Sustainable development of aquaculture depends strongly on the ability to control the epidemics that can impact farms [1]. Viral nervous necrosis (VNN), also called viral encephalopathy and retinopathy (VER), is caused by the single-stranded positive-sense RNA nervous necrosis virus (NNV) and is a major threat to the marine and freshwater fish farming industry, worldwide. It is responsible for increased mortality and reduced feed conversion ratio and animal welfare, thus affecting both production and economic viability of farms [2]. The European sea bass is one of the many species that are affected by this disease [3]. Because a warm environment is compatible with the occurrence of VNN [3–6], European sea bass can be highly impacted by this virus in the Mediterranean area and can devastate an entire production of susceptible larvae and juveniles, with a mortality rate that can reach 90% or more, while adults can also be impacted [3, 7]. Infected fish display abnormal swimming behavior [8], which is the main clinical sign, but they also show severe neurological disorders due to intensive vacuolation of the retina and the nervous system [9], and a rapid mortality peak, usually on the 10th day post-infection [10].

Several vaccination studies have been conducted for this disease, with very positive recent results [11] but these vaccines cannot yet be applied on larvae or early juveniles, for which this approach remains a challenge [12, 13]. Genetic improvement is another potential approach to improve disease resistance in aquaculture, in general [14], and specifically VNN resistance [15]. Recent studies have shown that European sea bass exhibits genetic variation in resistance to VNN with a heritability ranging from 0.26 to 0.43 [10, 16–19]. After validating the possibility to select and improve fish for resistance to NNV [14], dissection of the genetic architecture VNN resistance in several European sea bass populations is a necessary step to better understand the molecular architecture of infection-resistant fish and enable marker-assisted selection [20]. In this regard, genome-wide association studies (GWAS) have become a powerful tool and are commonly applied in animal breeding [21, 22]. Previously, GWAS for VNN resistance have been performed on European sea bass populations with 17K to 52K single nucleotide polymorphisms (SNPs) [10, 18, 23], and on Asian sea bass with 66K SNPs [19]. These studies reported the identification of quantitative trait loci (QTL) and SNPs that are associated with survival rate of sea bass exposed to NNV. In particular, a QTL was identified on linkage group (LG)12 in several populations of European sea bass [10, 18]. In these studies, the location of this QTL was, however, imprecise and population-dependent. Implementation of efficient and accurate marker-assisted selection requires the identification of the causative variant or tightly linked DNA variants. [24, 25]. The purpose of the present study was to identify candidate SNPs or other DNA variants for marker-assisted selection to improve resistance of European sea bass to VNN. To this end, we imputed whole-genome sequences on 5779 sea bass from two French breeding programs that were challenged with VNN and performed a GWAS with a Bayesian sparse linear mixed model (BSLMM) to identify the main genomic region of interest, which was then validated in an independent population.

## Methods

### Ethical statement

All infection challenges were carried out according to the European guidelines (Directive 2010–63-EU) and the corresponding French legislation. Animal experimental procedures were approved by the ethics committee on animal experimentation COMETH ANSES/ENVA/UPC No.16 and were authorized by the French Ministry of Higher Education, Research and Innovation under numbers 2017022816255366, 29/01/13-5 and 10/03/15-1.

### Populations and experimental design

#### Discovery populations

In total, 5799 European sea bass (*Dicentrarchus labrax*) from two commercial populations were used to identify QTL for resistance to VNN. These animals were produced by artificial mating and originated from two different French hatcheries designated as pop A and pop B. These populations are independent of each other but both originated from Mediterranean and Atlantic European sea bass (see Additional file 1: Fig. S1). The 2371 individuals from pop A and the 3428 individuals from pop B were distributed into six cohorts (3 for pop A, referred to as A_1, A_2 and A_3, and 3 for pop B, referred to B_1, B_2 and B_3). The A_1 and B_1 cohorts (see below) were previously studied in Griot et al. [10].

For pop A, the individuals from the three cohorts were not related and distributed as follows: 671 individuals in cohort A_1, derived from a partly factorial mating of 56 sires and 19 dams, 650 individuals in cohort A_2, derived from a partly factorial mating of 58 sires and 16 dams, and 1050 individuals in cohort A_3 derived from a partly factorial mating of 60 sires and 20 dams. Due to budget constraints, only the 174 sires (not the dams) of these individuals were sampled for genome sequencing. For pop B, cohorts B_1 and B_2 were related and B_3 was more distantly related. Cohort B_1 included 1083 individuals derived from a partly factorial mating of 40 sires and 14 dams, cohort B_2 included 1087 individuals derived from a partly factorial mating of 41 sires and 15 dams, and cohort B_3 included 1258 individuals derived from a partly factorial mating of 48 sires and 27 dams. For pop B, all parents of cohorts B_2 and B_3 were sampled for sequencing, while only some pairs of parents were sampled for cohort B_1 but only one parent (mostly the mothers) provided enough DNA to be sequenced for some individuals. Thus, for pop B, 159 parents were sampled for whole-genome sequencing. Full information is in Table 1.

**Table 1:** Description of the two commercial populations and the validation population (IFREMER) that were challenged with VNN

Population	Population A			Population B			IFREMER
Cohorts	A_1	A_2	A_3	B_1	B_2	B_3	Validation
Average weight	8	6	25	31	7	11	8
Average survival rate (%)	65.4	76.3	44.6	58.7	66.5	38.6	57.7
Number of individuals challenged and genotyped	1146	765	1148	1150	1246	1480	1536
Number of individuals challenged and genotyped passing filters	1015	652	1051	1096	1182	1293	1520
Number of parents (Sire/Dam)	56/19	58/16	60/20	40/14	41/15	48/27	4/4
Number of parents sequenced (Sire/Dam)	56/0	58/0	60/0	20/9	41/15	48/26	4/4
Number of individuals genotyped with sequenced parents	671	650	1050	1083	1087	1258	1334
Total of individuals genotyped with sequenced parents per population	2371			3428			1334

The fish from the cohorts were challenged with VNN on the SYSAAF-ANSES Fortior Genetics platform (ANSES, Plouzané, France). For all challenges, fish were maintained in filtered seawater in a flow-through system at a temperature of 27 ± 2 °C. Infectious challenges were performed separately but followed the same procedure for all cohorts, i.e. in tanks of the same size (400 L) and with a density below 35 kg/m^3^. All fish used for the challenge were individually tagged with RFID glass tags and acclimatized for a three-week period before the challenge. Infection with the red-spotted grouper nervous necrosis virus (RGNNV) strain W80 was performed during 4 h in a static bath of aerated seawater containing 1 × 10^5^ TCID_50_/mL of the virus. Mortality was recorded daily during the challenge period of on average 32 days. Fish that died during the challenge were categorized as susceptible and those that survived were considered resistant. To confirm the presence of the NNV virus in fish that died and eliminate the possible impact of unwanted bacterial coinfections, bacteriological and viral analyses were performed before the challenges and during peak mortality. Virologic analyses consisted of injecting a homogenized mixture of eye and brain sampled from a random dead fish on SSN1 (Striped Snakehead fish; *Ophicephalus striatus*) cells [26]. After 5 days of incubation, when cytopathic effects were observed on the cells, virus identification was performed by immunofluorescence using anti-NNV antibodies. For bacteriological analyses, spleen and kidney were sampled from dead fish and streaked on culture plates. The isolated bacteria were then identified by matrix-assisted laser desorption/ionization time-of-flight (MALDI-TOF) mass spectrometry.

#### Validation population

For the validation population, four full-sib families that were previously experimentally challenged with VNN, as described by Griot et al. [10] (NEM10, NEM12, SEM8 and WEM18), were used. The challenge method was the same described above. In total, 1536 fish from these families were challenged with NNV during 32 days. All eight parents of these four families were sampled for whole-genome sequencing.

### Whole-genome sequencing and variant calling

In total, pectoral fin biopsies of 333 parents of individuals that were challenged with VNN were collected to obtain whole-genome sequencing (WGS) data, including 174 parents from pop A and 159 parents from pop B. The eight parents of the validation population were also sequenced (see details in Table 1). Genomic DNA was extracted using the standard phenol–chloroform protocol at the GeT-PlaGe core facility, INRAE Toulouse, to perform DNA sequencing. DNA-seq libraries were prepared according to manufacturer’s protocols using the Illumina TruSeq Nano DNA HT Library Prep kit. Briefly, DNA was fragmented by sonication, size selection was performed using SPB beads (kit beads), and adaptors were ligated for traceability and sequencing. Library quality was assessed using an Advanced Analytical Fragment Analyzer and libraries were quantified by quantitative PCR using the Kapa Library Quantification kit. DNA sequencing was performed on an Illumina NovaSeq6000 using a paired-end read length of 2 × 150 bp with the Illumina NovaSeq6000 Reagent kits.

The resulting sequencing reads were aligned to the European sea bass reference genome that consists of 24 LG (seabass_V1.0) using the Burrows-Wheeler Aligner (BWA, v.2.1) method with default parameters [27]. SAMtools (v1.6) was used for handling SAM/BAM file formats [28] and duplicated reads were marked with the Picard tool (http://broadinstitute.github.io/picard v.2.21.1). After mapping, SNPs and InDels were called using the DeepVariant (v.1.1.0) tool [29] to retain all types of variants present in the analyzed populations. Next, the dataset for which SNPs were detected with a minimum read mapping quality of 30 and with coverage higher than 4 was kept using BCFtools (v.1.13) [28]. The second step of filtering was performed with the PLINK software (v.1.9) [30, 31] to remove SNPs that had: (1) a missing rate higher than 10%, (2) a minor allele frequency (MAF) lower than 1%, and (3) more than two identified alleles. Finally, the output VCF files were converted to PLINK format and SNP genotypes were coded as 0, 1, and 2, corresponding to the homozygous genotype for the reference allele from the published genome (seabass_V1.0), the heterozygous genotype, and the homozygous genotype for the alternative allele.

### SNP chip genotyping and imputation to whole-genome sequence

Samples from the challenged cohorts were genotyped for 56,730 SNPs using the ThermoFisher Axiom™ Sea Bass 57k SNP array DlabChip, at the Gentyane genotyping platform (INRAE, Clermont-Ferrand, France). In total, 3059, 3876, and 1536 individuals from pop A, pop B, and the validation population, respectively, were genotyped. Genotype calling was done using the ThermoFisher AxiomAnalysisSuite™ software. Preliminary quality controls were applied with cut-off values of 95% for SNP calling rate, 90% for sample calling rate, and the "Run PS Supplemental" option to regenerate SNP metrics to select the SNPs that were identified as polymorphic by the software. Two other filtering steps were applied to the genotypes of challenged individuals from pop A and B with the PLINK software (v.1.9) [30, 31], to remove (1) SNPs with a MAF less than 5% and (2) SNPs with a p-value for the Hardy–Weinberg test less than the 10^–8^ threshold. The output VCF files were converted to PLINK format and were coded as 0, 1, and 2, as described above for the sequencing data. Parentage assignment of the challenged fish to their sequenced parents was performed with the APIS R package [32] based on genotypes of a randomly sampled 1000 SNPs with a MAF around 0.5, with the positive assignment error rate set at 1%.

Imputation of the WGS SNPs to the offspring that were genotyped with the 57k SNP array DlabChip was performed using the FImpute software (v.3) [33], while accounting for pedigree information. The SNPs that were identified based on the WGS variant calling of the parents were used as reference and the 57K genotypes of the challenged offspring were used as target for pop A, pop B, and the validation population.

To reduce the number of high-effect SNPs in the association analyses of populations A and B, a subset of the imputed WGS SNPs was created from filters on linkage disequilibrium. Two successive filters were applied to have the same set of SNPs across the populations and to avoid redundancy among SNPs. First, we retained the SNPs that were shared among pop A, pop B, and the validation populations. Second, within each population, we filtered the remaining SNPs based on linkage disequilibrium (LD) between all pairs of SNPs, with an upper limit of r^2^ = 0.8 within 200-kb windows, using the PLINK software (v.1.9) [30, 31]. And finally, common SNPs after filtering on LD in each population were selected.

### Bayesian sparse linear mixed model for genome-wide association studies

The GWAS was conducted separately for each population by using a BSLMM that assumes that each of the SNPs has at least a relatively small effect but some SNPs may have a large effect [30, 31]. Therefore, BSLMM is capable of adapting to different genetic architectures of the studied trait, from the infinitesimal polygenic model to a model that assumes that only a very small proportion of all variants affect the phenotype. The general linear model that was fitted, separately for each population, to binary survival phenotypes adjusted for an estimate of the cohort effect obtained from a linear model (as recommended in the GEMMA manual to avoid the problem of covariates identical to some of the genotypes) can be presented as follows:

$$\mathbf{y}={\mathbf{1}}_{\mathbf{n}}\upmu +\mathbf{X}{\varvec{\upbeta}}+\mathbf{u}+{\varvec{\upvarepsilon}},$$

where $\mathbf{y}$ is the n-vector of adjusted survival phenotypes, ${\mathbf{1}}_{\mathbf{n}}$ is an n-vector of 1s, ${\mathbf{1}}_{\mathbf{n}}\upmu$ is a scalar representing the phenotype mean, $\mathbf{X}$ is an $\mathrm{n}\times \mathrm{p}$ matrix of genotypes measured on $\mathrm{n}$ individuals at $\mathrm{p}$ SNPs, ${\varvec{\upbeta}}$ is the corresponding $\mathrm{p}$-vector of the SNP effects; $\mathbf{u}$ is a vector of random additive genetic effects that are assumed distributed according to $N({\mathbf{0}}, \mathbf{K}{\upsigma }_{\mathrm{b}}^{2})$, where ${\upsigma }_{\mathrm{b}}^{2}$ the additive genetic variance and $\mathbf{K}$ is the genomic relationship matrix; and ${\varvec{\upvarepsilon}}$ is a $\mathrm{n}$-vector of residuals that are assumed distributed as $N({\mathbf{0}}, \mathbf{I}{\upsigma }_{\mathrm{e}}^{2})$, where ${\upsigma }_{\mathrm{e}}^{2}$ is the variance of the residual errors. Assuming $\mathbf{K}={\mathbf{X}\mathbf{X}}^{\mathbf{T}}/\mathrm{p}$, the SNP effects can be decomposed into two parts: a p-vector ${\varvec{\upalpha}}$, which captures the small effects that all SNPs have, and the p-vector ${\varvec{\upbeta}}$, which captures the additional effects of some large-effect SNPs. In the above model, $\mathbf{u}=\mathbf{X}{\boldsymbol{\upalpha}}$ can be viewed as the combined effect of all small-effect SNPs, and the total effect size for a SNP $\mathrm{i}$ is ${{\varvec{\upgamma}}}_{\mathrm{i}}={{\upalpha }}_{\mathrm{i}}+{\upbeta }_{\mathrm{i}}$. The individual SNP effects ${{\varvec{\upgamma}}}_{\mathrm{i}}$ were sampled from a mixture of two normal distributions, ${{\varvec{\upgamma}}}_{\mathrm{i}}\sim\uppi N(0,{\upsigma }_{\mathrm{\alpha }}^{2}+{\upsigma }_{\upbeta }^{2})+(1-\uppi )N(0,{\upsigma }_{\upbeta }^{2})$ where ${\upsigma }_{{\upalpha }}^{2}$ is the variance of the small SNP effects, ${\upsigma }_{\upbeta }^{ 2}$ is the additional variance associated with large SNP effects, and $\uppi$ is the proportion of SNPs with large effects.

The BSLMM was implemented using the genome-wide efficient mixed model association (GEMMA) software [34]. In GEMMA, we applied a linear BSLMM model using a Markov chain Monte Carlo (MCMC) method, as proposed by Zhou et al. [34]. In total, 5 million iterations (-s option) were performed with a burn-in of 100,000 and samples recorded every 10 iterations for further analysis. To ensure convergence of the distribution of the hyper-parameter π, the minimum and maximum numbers of SNPs that were sampled to be included in the model with large effects were set to 5 (-smin option) and 100 (-smax option), respectively. These threshold values were based on an initial run with default values that indicated that the median number of SNPs that had a large effect was about 20.

### QTL identification and annotation

The BSLMM uses a MCMC algorithm for sampling from the posterior distribution to obtain all parameters values, including SNP effect estimates $\widetilde{\beta }$, the hyper-parameter π, and a posterior inclusion probability (PIP) for each SNP that indicates the proportion of samples in which that SNP was classified as having a large effect. This proportion can be used for QTL mapping as it indicates the strength of the evidence that the SNP has a larger effect. Following Barbieri and Berger [35], regions whose SNPs have a PIP higher than 0.5 were selected (since SNPs from these regions are included in the model in most of the iterations). As a complementary approach, to define a less stringent threshold, Stephens and Balding [36] proposed to calculate a Bayes factor (BF) for a SNP as $\mathrm{BF}=\frac{\frac{\mathrm{PIP}}{(1-\mathrm{PIP})}}{\frac{\uppi }{(1-\uppi )}}$.

As proposed by Kass and Raftery [37], the natural logarithm transformation of the BF (logBF) was computed as twice the natural logarithm of the BF to produce values within the same range as the usual likelihood ratio test values, thus facilitating the determination of thresholds to declare QTL. A threshold logBF ≥ 10 was used to declare very strong evidence for a QTL, following Michenet et al. [38]. The BSLMM results were visualized via a Manhattan plot, with negative values for logBF set equal to 0. Peak SNPs were identified using the threshold logBF ≥ 10, showing strong evidence for a QTL. The credibility interval for the QTL included every SNP with a logBF > 5 within a 100-kb sliding window from the peak SNP. This procedure was repeated for each of the identified QTL regions.

To determine how QTL regions affected VNN resistance, we analyzed survival of challenged bars as a function of the genotype of SNPs located in those regions. For this purpose, we standardized the survival rate in each cohort to a survival rate of 50% after a probit transformation to allow all cohorts to be compared. Then, the percent survival of each genotype for the peak SNP was recalculated for each cohort. Finally, survival rates of the studied SNPs were averaged by cohort to facilitate discussion of the results. In addition, an ANOVA was performed using R to calculate statistically significant differences in survival between Resistant (R) and Susceptible (S) genotypes within a population (by cohort) and across the three populations.

Functional analysis of the identified QTL regions was performed by linking variants detected in the whole-genome sequences of the parents with the annotated reference genome assembly of the European sea bass. All annotated genes positioned within the QTL region were reported. To understand the role of the variants in the coding regions of the identified genes, all SNPs identified in the variant calling step were annotated based on the UCSC annotation [39] of the European sea bass reference genome with the SnpEff software (v.5.0) and default parameters [40]. SnpEff annotates variants in the VCF files based on their position and reference annotation. Each SNP was classified as coding or non-coding, the impact (from high to low) of each SNP was identified and, finally, each SNP was assigned to the functional class of non-synonymous or synonymous (silent).

## Results

### VNN phenotypes

**Fig. 1:**
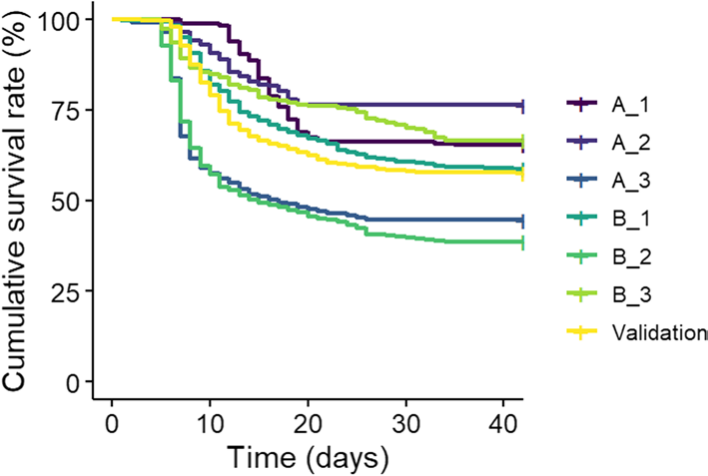
Evolution of the Kaplan–Meier cumulative survival to a VNN infection challenge for each cohort from two commercial populations and a validation population. Results are in purple for pop A (A_1, A_2, A_3), in green for pop B (B_1, B_2, B_3), and in yellow for the validation population

The six challenges for the six cohorts of pop A and B were conducted over approximately 32 days. Presence and absence of NNV and pathogenic bacteria were confirmed on several subsets of dead fish during the infection, validating NNV-related deaths. Survival rates ranged from 44.5 to 76.3% for the pop A cohorts and from 38.6 to 66.5% for the pop B cohorts. For the validation population, the average survival rate was 56% (Table 1). The peak of mortality was between 5- and 15-days post-infection for all cohorts (Fig. 1).

### From whole-genome sequences to imputed whole-genome variants

We generated WGS at an average coverage of 19-fold for 174 sires from pop A, 159 parents (126 sires and 53 dams) from pop B, and the eight parents (4 sires and 4 dams) of the validation population. Variant calling identified around 8 million raw variants. Quality control of the WGS SNPs was first carried out by population. All sequenced individuals were retained. The number of SNPs removed based on MAF (less than 15%), SNP call rate (less than 95%), and Hardy-Weindberg (p-value > 10^–8^) filters are reported in Additional file 2: Table S1. In total, 2,506,457 and 2,436,692 SNPs were retained for pop A and B, respectively.

From the 5799 57K genotypes obtained for pop A and B, respectively, 688 and 277 animals with a call rate threshold less than 90% or based on pedigree data were removed, i.e. keeping only individuals that were challenged and that had at least one parent sequenced. After SNP filtering based on MAF (less than 10%), call frequency (less than 95%), and the Hardy–Weinberg equilibrium, 40,743 SNPs and 36,862 SNPs were retained for pop A and B, respectively. Thus, genotypes of 2371 pop A animals for 40,743 SNPs and genotypes of 3428 pop B animals for 36,862 SNPs were retained for further analyses (for more details see Additional file 2: Table S1).

After the imputation step for pop A and B, we obtained genotypes for 2,506,457 and 2,436,691 imputed WGS SNPs for 2371 and 3428 challenged animals in pop A and pop B, respectively. Based on an r^2^ > 0.8 in 200-kb windows, 1,457,298 WGS SNPs and 1,457,105 WGS SNPs were retained for pop A and pop B, respectively. Finally, to perform GWAS on pop A and B, a common subset of SNPs was created after applying LD filters to each population, which included 838,451 imputed WGS SNPs.

For the validation population, the same filters as described above were used. For the WGS of the eight parents, 2,392,123 WGS SNPs were retained. For the 57K genotyping data of the challenged individuals, genotypes of 1334 individuals for 43,004 SNPs were retained. The imputation step resulted in a genetic marker set of 2,392,123 WGS imputed SNPs for the challenged validation population individuals.

### QTL detection for VNN resistance

**Fig. 2:**
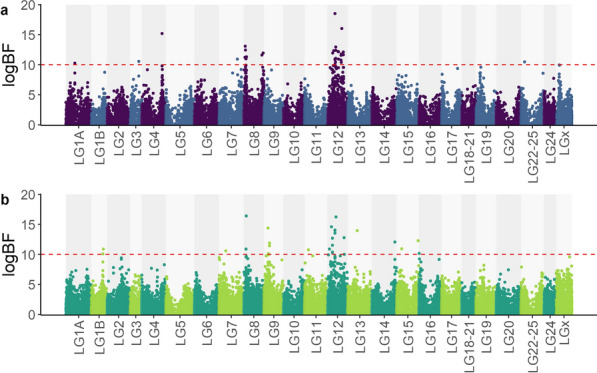
Manhattan plots of for VNN resistance in European sea bass populations A (**a**) and B (**b**). The red dashed line represents the logBF threshold of 10, corresponding to strong evidence for the presence of a QTL

The estimates of the genomic heritability of binary survival on the observed scale were 0.22 in pop A and 0.21 in pop B, combining the three cohorts for each population. The estimated proportion of genetic variance explained by the polygenic component was 61% for pop A and 62% for pop B. The BSLMM association analysis identified 11 QTL regions for pop A and 12 QTL regions for pop B as having large effects on VNN resistance, with strong evidence for these QTL (Table 2). In pop A, QTL were detected on LG1A, LG3, LG4, LG7, LG8, LG12, LG15, LG22-25, and LGX. In pop B, QTL detected were located on LG1B, LG7, LG8, LG9, LG12, LG13, LG14, and LG15 (Fig. 2).

**Table 2:** QTL regions identified in the GWAS, ranked by their position on the genome

QTL-ID	LG	Start (bp)	End (bp)	PIP	logBF	Population
LG1A_QTL	LG1A	10,107,908	10,111,686	0.02	10.3	pop A
LG1B_QTL	LG1B	13,152,351	13,885,885	0.04	10.9	pop B
LG3_QTL	LG3	10,137,843	10,137,843	0.03	10.6	pop A
LG4_QTL	LG4	23,334,300	23,618,451	0.22	15.2	pop A
LG7_QTL_1	LG7	7,828,421	7,828,421	0.03	10.6	pop B
LG7_QTL_2	LG7	21,062,741	21,062,741	0.03	10.9	pop A
LG8_QTL_1	LG8	1,423,728	3,198,660	0.38	16.4	pop A/pop B
LG8_QTL_2	LG8	20,789,708	22,055,049	0.05	12	pop A
LG9_QTL	LG9	4,532,879	6,335,544	0.18	14.4	pop B
LG12_QTL_1	LG12	4,717,307	7,246,406	0.2	14.6	pop A/pop B
LG12_QTL_2	LG12	8,671,217	8,797,942	0.59	18.5	pop A/pop B
LG12_QTL_3	LG12	9,118,505	9,821,502	0.36	16.2	pop A/pop B
LG12_QTL_4	LG12	12,167,000	19,133,722	0.3	16	pop A/pop B
LG13_QTL	LG13	11,019,998	11,019,998	0.15	14	pop B
LG14_QTL	LG14	26,274,292	26,276,196	0.07	12.1	pop B
LG15_QTL	LG15	5,885,758	5,885,758	0.04	11	pop B
LG15_QTL	LG15	24,678,361	24,678,361	0.07	12.3	pop B
LG22-25_QTL	LG22-25	4,185,377	4,185,377	0.03	10.5	pop A

*TL-ID* QTL identifiers, *LG* linkage group, *bp* base pair, *PIP* posterior inclusion probability, *logBF* logarithm of Bayes Factor, *Population* population identification of the QTL region

When the results for pop A and B were compared, several significant SNPs with a strong effect for both populations were detected on LG8 and LG12. On LG8, the QTL region between 1,423,728 bp and 3,198,660 bp (LG8_QTL_1) was shared between the two populations. This QTL explained 2.6% of the total genetic variance in pop A and 3.1% in pop B. Based on logBF, four shared QTL regions were identified on LG12: LG12_QTL_1 between 4,717,307 pb and 7,246,406 pb, LG12_QTL_2 between 8,671,217 pb and 8,797,942 pb, LG12_QTL_3 between 9,118,505 pb and 9,821,502 pb and LG12_QTL_4 between 12,167,000 pb and 19,133,722 pb. The 126,725-pb long LG12_QTL_2 QTL region had a major effect in both populations, with the major SNP, at 8,797,936 pb, explaining 21.8% of the total genetic variance in pop A and 20.3% in pop B. For all the other shared QTL regions on LG12, the major SNPs explained only a small proportion of the genetic variance (less than 2%).

### LG12 QTL effect and candidate genes

Survival rates for the genotypes at the SNP with the highest logBF value were calculated for the LG12_QTL_2 for both commercial populations and in the validation population.

**Fig. 3:**
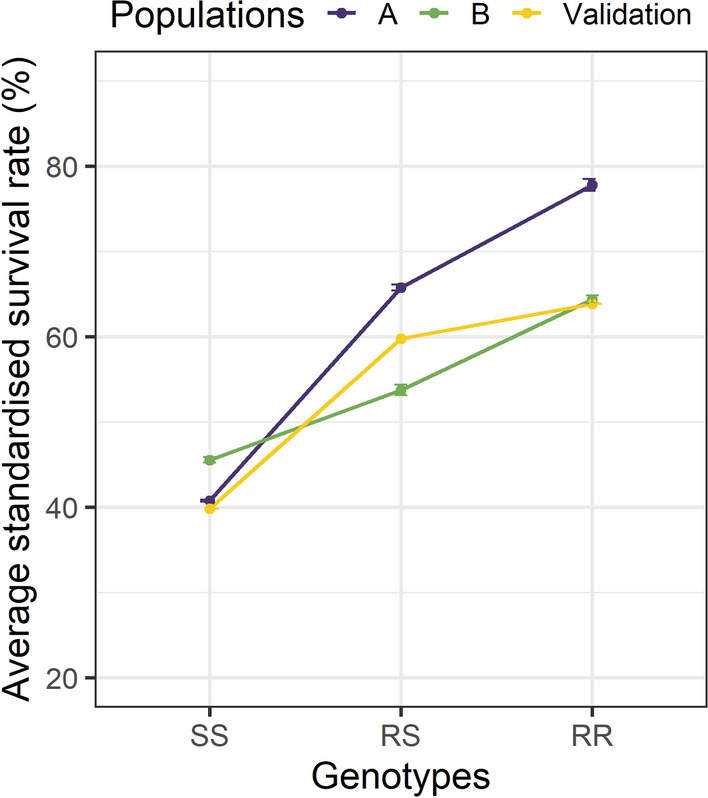
Average standardised survival rate for the peak SNP in the LG12_QTL_2. Each point corresponds to a weighted mean of the average survival rate found in each cohort. The standard deviation of survival among the three cohorts is reported for each population

In Fig. 3, the resistant genotypes at the peak SNP for LG12_QTL_2 (LG12_8797936) are annotated “RR”, “RS” and “SS” for resistant, heterozygous, and susceptible genotypes, respectively, which is the major SNP. At this SNP, the reference allele *G* corresponds to the resistant allele (R) and the alternative allele *T* is the susceptibility allele (S). The flanking sequences of the LG12_8797936 SNP for the reference and alternative alleles are in Additional file 3. In pop A, the average survival rate was 78% for the resistant genotype and 40.6% for the susceptible genotype. In pop B, resistant and susceptible genotypes were associated with average survival rates of 66.2 and 45.4%, respectively. Survival rates were very similar in the validation populations, i.e. 39.9% for the susceptible genotype and 63.8% for the resistant genotype. An ANOVA analysis was performed on the R and S genotypes of the LG12_8797936 SNP regarding the VNN resistance for pop A, pop B, and the validation population. The average standardized survival for each population is shown in Fig. 3 and Table 3 shows the statistical differences between genotypes for each cohort. For the other QTL regions identified on LG12, the peak SNP showed a significant impact on survival rate only in the population in which it was detected, especially for QTL_LG12_1 and QTL_LG12_3 (data not shown).

**Table 3:** ANOVA for the effect of the genotype at the LG12_8797936 variant on VNN resistance for each population and cohort

Population	Cohort	N	df	Mean squares	P value	Statistical significance
Pop A	–	2371	2	14.85	4.22E−28	***
	A_1	671	2	5.55	9.65E−12	***
	A_2	650	2	3.00	4.34E−08	***
	A_3	1050	2	7.31	6.41E−14	***
Pop B	–	3428	2	0.77	4.53E−02	*
	B_1	1083	2	6.20	4.53E−12	***
	B_2	1258	2	0.17	4.61E−01	
	B_3	1087	2	1.14	8.04E−03	**
Validation	–	1334	2	6.81	4.53E−13	***

*N* number of European seabass, *df* degree of freedom

Statistical significance = significant codes 0: ‘***’ < 0.001: ‘**’ < 0.01: ‘*’ < 0.05: ‘.’ < 0.1: ‘’ < 1; Cohort = code "-" corresponds to full populations, i.e. including the three cohorts

**Fig. 4:**
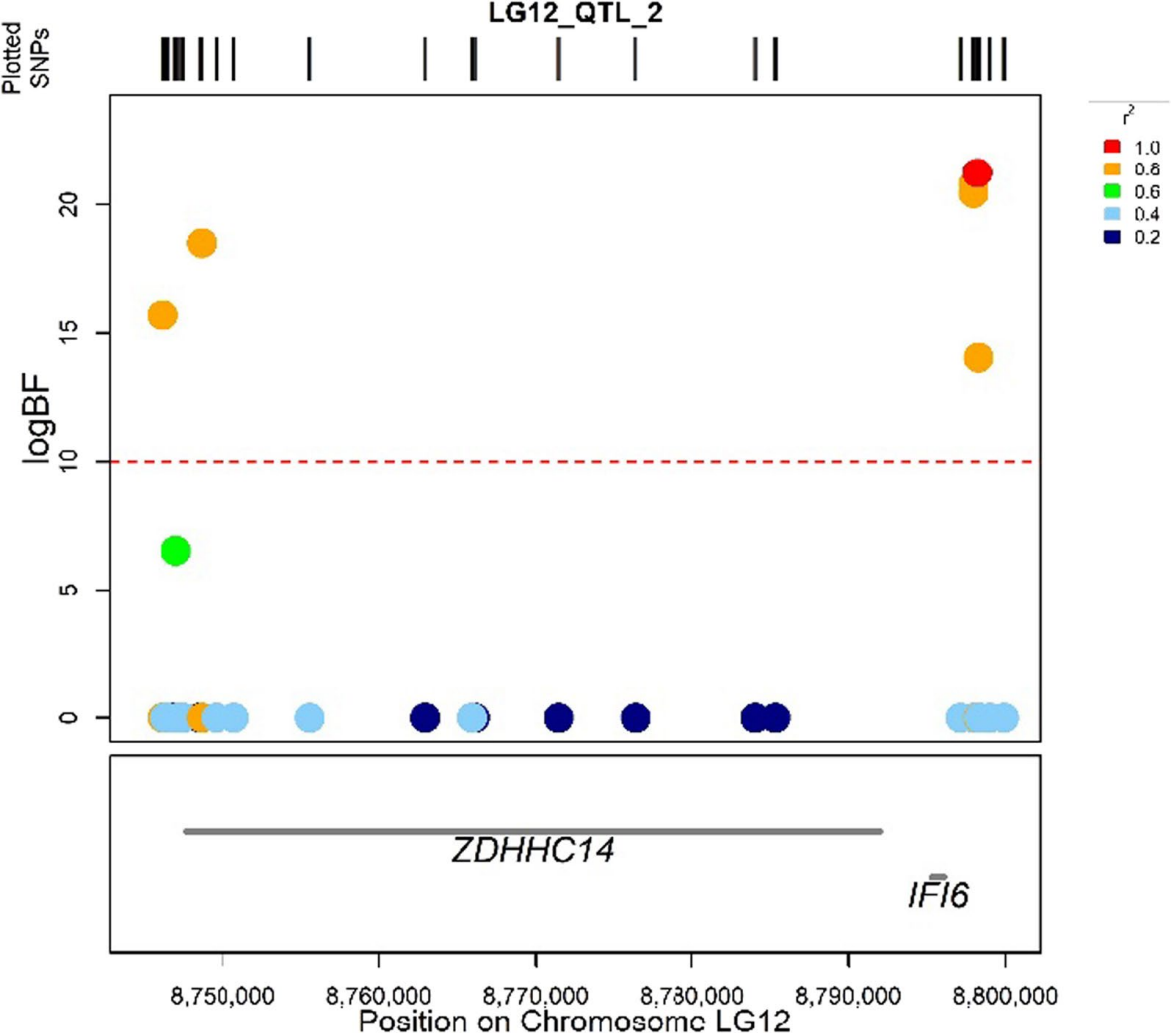
Results for the LG12_QTL_2 region showing the SNPs with their genomic positions and the identified genes. The graph displays the logBF value on the y-axis and the physical position on the x-axis. Each point represents a variant in the QTL region, colored according to their LD (based on the squared coefficient of correlation, r^2^ measure) with the characteristic variant of this QTL (red point). The red dashed lines indicate the logBF = 10 threshold

Based on the UCSC genome annotation database, two genes were located in the LG12_QTL_2 region (Fig. 4), i.e. *IFI6/IFI27-like*, which encodes an interferon alpha-induced protein, and *ZDHHC14,* which encodes a zinc finger palmitoyltransferase. The *IFI6/IFI27-like* gene is 903 pb long with four exons and the *ZDHHC14* gene is 44.4 kb long with nine exons, respectively, and they are at 1.9 and 6 kb from the SNP with the highest logBF of the LG12_QTL_2.

Analyses of the SNPs in the LG12_QTL_2 region did not identify causal variants that impact the coding region of the *IFI6/IFI27-like* gene since all SNPs are positioned in non-coding downstream sequences, in particular the LG12_8797936 SNP with the strongest effect in the GWAS. For the *ZDHHC14* gene, LG12_8746942 was found to be the SNP most strongly associated with the resistance phenotype and the SnpEff approach showed that it is located within the coding sequence of the *ZDHHC14* gene, but with no impact on protein structure.

## Discussion

In the present study, our aim was to use a combination of whole-genome sequencing, 57K genotyping, and GWAS approaches to identify QTL for VNN resistance in European sea bass and to characterize the QTL using public functional annotation. Our results show that heritability estimates for VNN survival using imputed genotypes (0.21–0.22 on the observed scale) are of the same magnitude as those obtained in previous studies on NNV-infected sea bass, i.e. 0.08 to 0.23 on the observed scale in [10, 23] and 0.15 to 0.43 on the liability scale in [18, 41]. The differences between the estimates of NNV survival in sea bass among all these studies can be due to differences in the number of breeders used to obtain challenged individuals, the number of experimental individuals, the method of infection during viral challenges, the age and size at challenge, the genetic origin of the fish, and the experimental design [41].

Previous studies on the genetic architecture of VNN resistance in European sea bass revealed several genomic regions with one or more significant SNPs. The majority of the previously reported QTL were based on a defined chromosome-wide significance threshold. Based on RAD sequencing, Palaiokostas et al. [18] identified QTL on chromosomes 3, 20, and 25 but the nomenclature that they used does not match with the LG nomenclature used in our study. However, after performing Blast on the European sea bass reference genome (seabass_V1.0) for these QTL regions, we were able to localise them on LG6 (for which we found no QTL) and LG12 (for which we found three QTL). Based on the use of the 57k SNP array DlabChip and a Bayesian approach, Griot et al. [10] identified, in pop A and B, seven QTL regions (LG3, LG8, LG14, LG15, LG19 and LG20) and a large QTL on LG12, and this large QTL region on LG12 was confirmed in three of the four experimental families in the validation population with an interval mapping approach. One highly significant QTL on LG12 was reported to be shared between pop A and B. More recently, Vela-Avitúa et al. [23] also identified a QTL with a strong effect on LG12 within a 7-Mb region on a different population, using the same 57k SNP array DlabChip. Thus, LG12 seems to be of special interest as a QTL was found on this chromosome in all studies and populations, except in one experimental family in [10].

Whole-genome sequencing of the parents of 57K genotyped offspring was used to finely map SNPs and InDels to identify candidate genes for VNN resistance. Whole-genome sequence variants contain more causal mutations than the SNP genotyping arrays that are available for European sea bass, which means that our study is more likely to detect causal mutations. The relevance of using sequence data for mapping traits in farm animals was shown in the 1000 bull genomes program [42]. We conducted whole-genome association studies for VNN survival using imputed sequence data and Bayesian fine mapping was performed to accurately map candidate variants for this binary trait. Several QTL regions were identified in the two commercial populations studied, but with most QTL regions revealed only in one of the two populations. Indeed, when we studied the survival rates associated with the genotypes of the peak SNPs in these regions, they affected VNN survival only in the population in which they were detected. The only peak SNP that had a strong association with survival in both populations was the LG12_8797936 SNP in the LG12_QTL_2. The effect of this SNP was further validated using the unrelated validation population, which also showed a fairly significant effect on survival rate associated with the resistant and susceptible genotypes at this SNP (Fig. 3). The LG12_8797936 SNP that characterizes this QTL region was associated with a survival rate that increases from 40% for the susceptible genotype to nearly 80% for the resistant genotype in pop A, thus revealing a strong impact of this QTL. In pop B, the effect of this SNP on survival rates was slightly less strong, at 45 and 64% for the susceptible and resistant genotypes, respectively. In the validation population, the survival rates for the susceptible and resistant genotypes were 40 and 64%, respectively. For the other SNPs present in the 127kb LG12_QTL_2, the survival rates were not significantly different between genotypes at the SNP for all three populations, whereas SNP LG12_8797936 showed a significant difference in survival rate compared to genotypes for commercial populations studied (Pop A and Pop B). In contrast, the two QTL that were identified at each end of LG12 appeared to be characteristic of a single population. Thus, our focus was on the genomic region shared by both pop A and B.

Finding the causal variants that underlie a QTL is a very difficult task, and very few causal variants have been identified to date. Using sequence data, we were able to refine the QTL region associated with VNN resistance and identify the genes located in this region, although prioritization of the putative causal genes is challenging with the current state of annotation of the European sea bass genome, which counts 23,382 coding genes and 35,707 transcribed genes [39]. In spite of these limitations, the genes that are located within the QTL region on LG12, i.e., the 127-kb region that spanned the most significant SNPs, were searched using the European sea bass genome (seabass_V1.0 [39]) and two genes separated by 3.6 kb, *ZDHHC14* and *IFI6/IFI27-like* were identified as strong candidates. Neither of these genes were reported in previous association studies for survival to VNN. Interestingly, LG12_QTL_2 and these two genes are located more than 2.5 Mb away from the QTL region identified by Vela-Avitúa et al. [23], which includes the *HSP70* gene. This may be a consequence of the much higher precision of our localization, as the LG12_QTL_2 region is only 127 kb long, while the region identified in [23] was 7 Mb long. Another difference between our study and that of [23] is the way the infectious challenge was performed, with the latter infecting fish by injection, while we induced infection via contamination of the circulating water, i.e. the virus was present in the aquatic environment, as is the case for the fish in rearing facilities (tanks, sea cage). As a result, the defence mechanisms involved in the immune response may differ between the two studies. Interestingly, the *HSP70* gene was also as identified differentially expressed in a study that compared NNV infected and non-infected cell lines of European sea bass [43]. This study identified genes that are triggered by infection but these genes are not necessarily those involved in disease resistance.

As for the variants identified in our study, the LG12_8746942 SNP is located in the coding region of the *ZDHHC14* gene, which encodes a palmitoyltransferase that can catalyze the addition of palmitate onto various protein substrates. A recent study linked this reaction with inhibition of NNV in vitro and showed that inhibition of protein palmitoylation and phospholipid synthesis, which involve lipid metabolism, significantly decreased RGNNV replication [43], noting that viruses hijack host cell metabolism to ensure their own replication. However, in vitro experiments showed that zinc finger DHHC domain–containing palmitoyltransferases can enhance the activity of interferon-induced transmembrane protein 3 (IFITM3), which is a cellular endosome- and lysosome-localized protein that restricts numerous virus infections, and its activation by ZDHHC has antiviral properties [44]. In addition to the lesions in the liver and spleen tissues, lipid droplets present in the necrotic nerve cells are used by the virus for replication [15]. As these symptoms occur in sea bass during infection with NNV, the *ZDHHC14* gene is of great interest and deserves to be analyzed further via functional analyses.

The SNP with the strongest effect on the survival rate in the LG12_QTL_2 region is 3.7 kb downstream from the *IFI6/IFI27-like* gene that encodes an interferon-inducible protein. In most cells, interferon response is a major first line of defense against viral infection. Viral infection triggers the production of interferon, which binds to ubiquitously expressed receptors on nearby cells and induces a powerful transcriptional program that comprises hundreds of interferon-stimulated genes [45]. Initially discovered in chicken, interferon and its essential role in orchestrating the immune response to viral infection have since then been identified in all vertebrates, including in fish [46]. The *IFI6/IFI27-like* gene, which encodes a small 130 amino acid interferon-inducible protein from the IFI6 family and has four conserved paralogues in humans (*IFI6*, *IFI27*, I*FI27L1* and *IFI27L2*), is involved in several processes of the regulation of human viral attacks in response to type I interferon (I-IFN) [47, 48]. For hepatitis B virus (HBV) replication, in vivo analysis based on the combined injection of an *IFI6/IFI27-like* expression plasmid and HBV revealed significant inhibition of HBV DNA replication and gene expression [49]. The same mechanism was also reported for the hepatitis C virus [50]. In several fish species, including Atlantic halibut, turbot, grouper, Asian sea bass, and European sea bass [51–55], VNN infection induces expression of interferon and interferon-related genes. In particular, I-IFN was shown to reduce RGNNV replication and thus protect fish against this virus in grouper fish [51, 55]. Based on these results, a similar study on European sea bass identified the I-IFN system as an important player in its protection against RGNNV infection using different in vivo approaches [52]. Thus, based on the results obtained on the regulation of viral infections in humans but also in several aquaculture species, resistance to NNV in sea bass could be related to the interferon pathway via the regulation of the IFI6/IFI27-like protein encoded by the gene positioned on LG12. Additional functional approaches need to be conducted on both the *IFI6/IFI27-like* and *ZDHHC14* genes to evaluate their impact on immune mechanisms of European sea bass against VNN.

## Conclusions

We identified candidate genes associated with resistance to VNN in European sea bass using sequence data GWAS. Based on dense genotypic information, the BSLMM approach was performed to refine the GWAS. Among the identified putative QTL, two were shared between the two commercial populations that were studied, among which QTL LG12_QTL_2 that carries the *IFI6/IFI27-like* and *ZDHHC14* genes, the role of which should be further clarified using other functional approaches. For practical application in selective breeding, the LG12_8797936 SNP identified in this QTL and located near the *IFI6/IFI27-like* gene, has a high potential for successful marker-assisted selection, since we show that the susceptible and resistant genotypes had a similar effect on survival to VNN infection in independent populations. This is a major advance to improve the resistance of cultured populations of European sea bass to one of its main infectious diseases.

## Supplementary Information

**Additional file 1: Figure S1.** PCA on genotypes of commercial populations (pop A and pop B) and wild populations in the Atlantic (Atl) and Eastern Mediterranean (East-Med). Principal component analysis (PCA) using genotypic data from two commercial populations and two wild populations, one from the Atlantic strain (Atl) and the other from the West Mediterranean strain (East-Med).

**Additional file 2: Table S1.** Number of SNPs removed after several quality filters. Details of the number of SNPs removed with quality controls applied on whole-genome sequences and 57K SNP chip data.

**Additional file 3:** Flanking 50-bp sequences for the top associated SNPs in LG12_QTL_2. Genomic sequences flanking the *IFI6/IFI27-like* and *ZDHHC14* SNPs computed from *Dicentrarchus labrax* genome (seabass_V1.0).

## Data Availability

Individual whole-genome sequencing data from the parents of the validation population are available from NCBI SRA under the BioProject access code PRJNA915039 (https://www.ncbi.nlm.nih.gov/sra/PRJNA915039). The other datasets used and/or analyzed during the current study are available for scientific purposes from the corresponding author upon reasonable request.
