# Supplementary material for: Whole‐genome sequencing identifies interferon-induced protein IFI6/IFI27-like as a strong candidate gene for VNN resistance in European sea bass
Source: Genet Sel Evol. 2023 May 4;55:30. doi: 10.1186/s12711-023-00805-2 (PMC10161657; doi:10.1186/s12711-023-00805-2)

**Additional file 1: PCA on genotypes of commercial populations (pop A and pop B) and wild populations in the Atlantic (Atl) and Eastern Mediterranean (East-Med)**


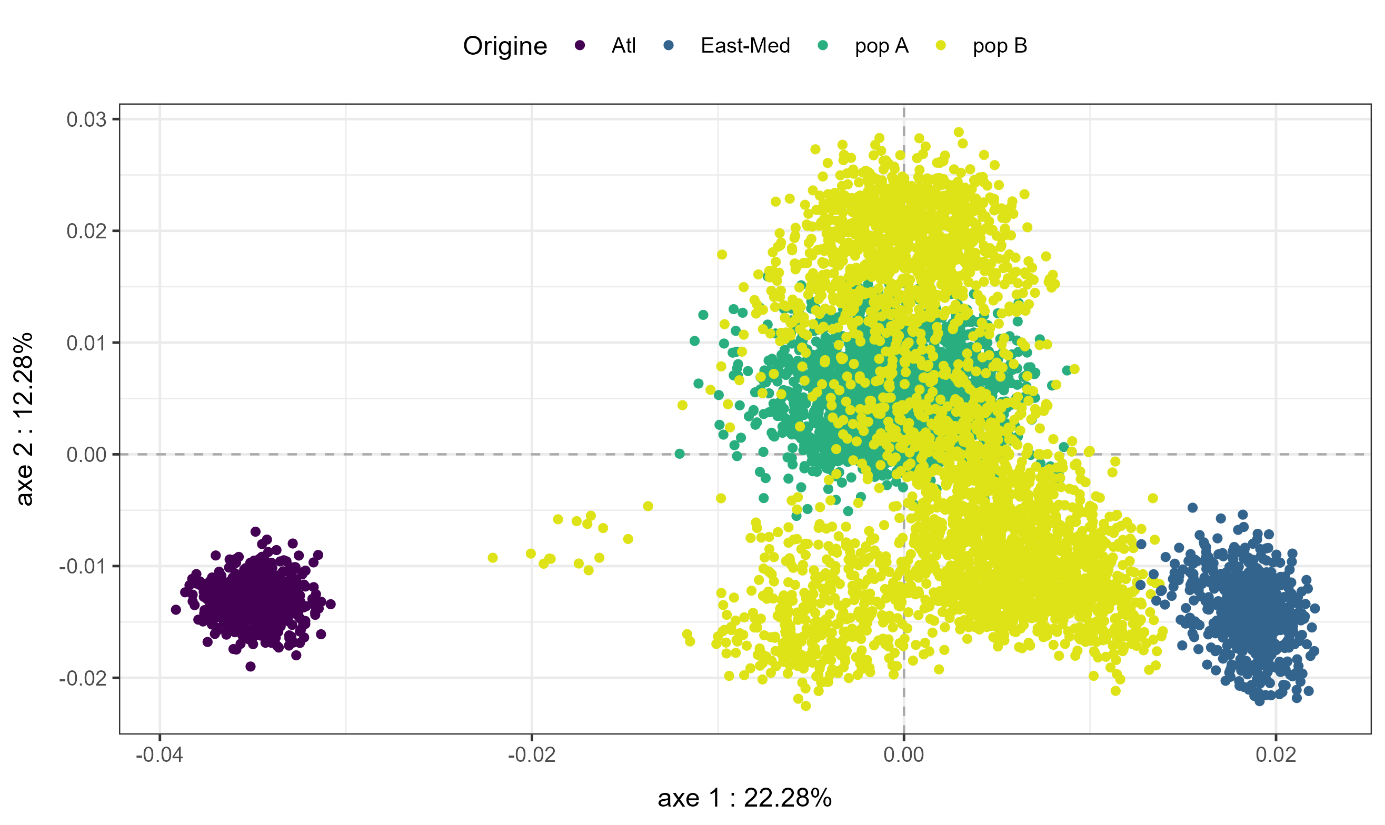

Supplement: Supplementary file 1 — Additional file 1: Figure S1. PCA on genotypes of commercial populations (pop A and pop B) and wild populations in the Atlantic (Atl) and Eastern Mediterranean (East-Med). Principal component analysis (PCA) using genotypic data from two commercial populations and two wild populations, one from the Atlantic strain (Atl) and the other from the West Mediterranean strain (East-Med). [file 12711_2023_805_MOESM1_ESM.docx]
