# Supplementary material for: Whole‐genome sequencing identifies interferon-induced protein IFI6/IFI27-like as a strong candidate gene for VNN resistance in European sea bass
Source: Genet Sel Evol. 2023 May 4;55:30. doi: 10.1186/s12711-023-00805-2 (PMC10161657; doi:10.1186/s12711-023-00805-2)
Supplement: Supplementary file 3 — Additional file 3: Flanking 50-bp sequences for the top associated SNPs in LG12_QTL_2. Genomic sequences flanking the IFI6/IFI27-like and ZDHHC14 SNPs computed from Dicentrarchus labrax genome (seabass_V1.0). [file 12711_2023_805_MOESM3_ESM.docx]

**Additional file 3:** **Flanking 50-bp sequences for the top associated SNPs in LG12_QTL_2**

- **LG12:8746917-8746967** with LG12_8746942 SNP close to ZDHHC14 gene:

CCAAAGCTTAACTAGGAGAGGGGCC**[C/T]**GTGAGGCCTCGGGATCGGGGTGCTG

- **LG12:8797911-8797961**with the LG12_8797936 SNP close to IFI6/IFI27-like gene:

CTTTACACACAGTTTCTTGGTGTTG**[G/T]**GCTGCTGACAATATATAACTTTCAC
